# Bioinformatic analyses in early host response to Porcine Reproductive and Respiratory Syndrome virus (PRRSV) reveals pathway differences between pigs with alternate genotypes for a major host response QTL

**DOI:** 10.1186/s12864-016-2547-z

**Published:** 2016-03-08

**Authors:** Martine Schroyen, Christopher Eisley, James E. Koltes, Eric Fritz-Waters, Igseo Choi, Graham S. Plastow, Leluo Guan, Paul Stothard, Hua Bao, Arun Kommadath, James M. Reecy, Joan K. Lunney, Robert R. R. Rowland, Jack C. M. Dekkers, Christopher K. Tuggle

**Affiliations:** Department of Animal Science, Iowa State University, 2255 Kildee Hall, Ames, IA 50011 USA; Department of Statistics, Iowa State University, 1121 Snedecor Hall, Ames, IA 50011 USA; Department of Animal Science, University of Arkansas, AFLS B106D, Fayetteville, AR 72701 USA; USDA-ARS, BARC, APDL, Bldg.1040, Beltsville, MD 20705 USA; Department of Agricultural, Food and Nutritional Science, University of Alberta, Edmonton, AB T6G 2P5 Canada; College of Veterinary Medicine, Kansas State University, K-231 Mosier Hall, Manhattan, KS 66506 USA

**Keywords:** Pig, PRRS virus, RNA-seq, WUR, GBP5, Ion transport, Homeostasis, Regulatory factors

## Abstract

**Background:**

A region on *Sus scrofa* chromosome 4 (SSC4) surrounding single nucleotide polymorphism (SNP) marker WUR10000125 (WUR) has been reported to be strongly associated with both weight gain and serum viremia in pigs after infection with PRRS virus (PRRSV). A proposed causal mutation in the guanylate binding protein 5 gene (*GBP5*) is predicted to truncate the encoded protein. To investigate transcriptional differences between WUR genotypes in early host response to PRRSV infection, an RNA-seq experiment was performed on globin depleted whole blood RNA collected on 0, 4, 7, 10 and 14 days post-infection (dpi) from eight littermate pairs with one AB (favorable) and one AA (unfavorable) WUR genotype animal per litter.

**Results:**

Gene Ontology (GO) enrichment analysis of transcripts that were differentially expressed (DE) between dpi across both genotypes revealed an inflammatory response for all dpi when compared to day 0. However, at the early time points of 4 and 7dpi, several GO terms had higher enrichment scores compared to later dpi, including inflammatory response (*p* < 10^-7^), specifically regulation of NFkappaB (*p* < 0.01), cytokine, and chemokine activity (*p* < 0.01). At 10 and 14dpi, GO term enrichment indicated a switch to DNA damage response, cell cycle checkpoints, and DNA replication. Few transcripts were DE between WUR genotypes on individual dpi or averaged over all dpi, and little enrichment of any GO term was found. However, there were differences in expression patterns over time between AA and AB animals, which was confirmed by genotype-specific expression patterns of several modules that were identified in weighted gene co-expression network analyses (WGCNA). Minor differences between AA and AB animals were observed in immune response and DNA damage response (*p* = 0.64 and *p* = 0.11, respectively), but a significant effect between genotypes pointed to a difference in ion transport/homeostasis and the participation of G-coupled protein receptors (*p* = 8e-4), which was reinforced by results from regulatory and phenotypic impact factor analyses between genotypes.

**Conclusion:**

We propose these pathway differences between WUR genotypes are the result of the inability of the truncated GBP5 of the AA genotyped pigs to inhibit viral entry and replication as quickly as the intact GBP5 protein of the AB genotyped pigs.

**Electronic supplementary material:**

The online version of this article (doi:10.1186/s12864-016-2547-z) contains supplementary material, which is available to authorized users.

## Background

Porcine reproductive and respiratory disease (PRRS), also known as mystery swine disease or blue ear disease, emerged in the late 80’s and 90’s and is one of the most economically important diseases affecting pigs worldwide [1]. The disease results in severe reproductive losses, such as late-term abortions and mummified and stillborn fetuses, as well as neonatal piglets developing severe dyspnea and tachypnea and demonstrating an increased morbidity and mortality rate [2]. In weaned pigs, PRRS manifests itself by pneumonia, lethargy, failure to thrive and a higher mortality rate, mainly due to the co-existence with other infections [2]. To date, production costs of PRRS are estimated at $664 million a year in the US alone [3].

Many efforts have been, and continue to be, made to understand the PRRS virus (PRRSV), its replication and the immune response evoked in the host [4–6]. Modified live-attenuated and inactivated vaccines against PRRSV are used widely, primarily to improve performance in PRRS positive herds, but often fail to elicit complete protection particularly against highly heterologous PRRSV isolates [7]. There therefore remains a need for novel strategies to combat PRRS including the development of cross-protective PRRSV vaccines. Moreover, long-distance airborne transport of PRRSV complicates control strategies even further [8]. The PRRS host genetics consortium (PHGC) was founded to investigate the potential for control of the disease from the host point of view [9]. Boddicker et al. [10] performed a Genome Wide Association Study (GWAS) on the first three PHGC trials with 600 infected weaner pigs and identified a region on *Sus scrofa* chromosome 4 (SSC4) surrounding a single nucleotide polymorphism (SNP) marker WUR10000125 (WUR) that was strongly associated with weight gain and viral load after a PRRS infection [10]. This association was validated and expanded to other pig crosses in an additional 5 PHGC trials [11, 12]. Compared to AB and BB animals, the AA animals for the WUR SNP had higher levels of viremia measured over 21 days post-infection (dpi) and a reduced growth rate over 42 dpi. No differences were seen between AB and BB animals, pointing to a dominant effect of the B allele [10]. Further examination of the region surrounding WUR revealed that guanylate binding protein 5 (*GBP5*) is a strong candidate gene, due to its differential expression during PRRSV infection, the presence of splice variant differences between AA and AB animals and its role in inflammasome assembly during immune response [13]. We hypothesized that the global gene expression can be impacted by the genotype which is associated with the different host response. In order to elucidate these differences, 8 littermate pairs, each with one AA and one AB animal, from PHGC trial 3 were selected for transcriptome analysis of blood collected up to 14 days post-infection using RNA-seq.

## Results

### Identification and annotation of differentially expressed (DE) transcripts over time

**Fig. 1:**
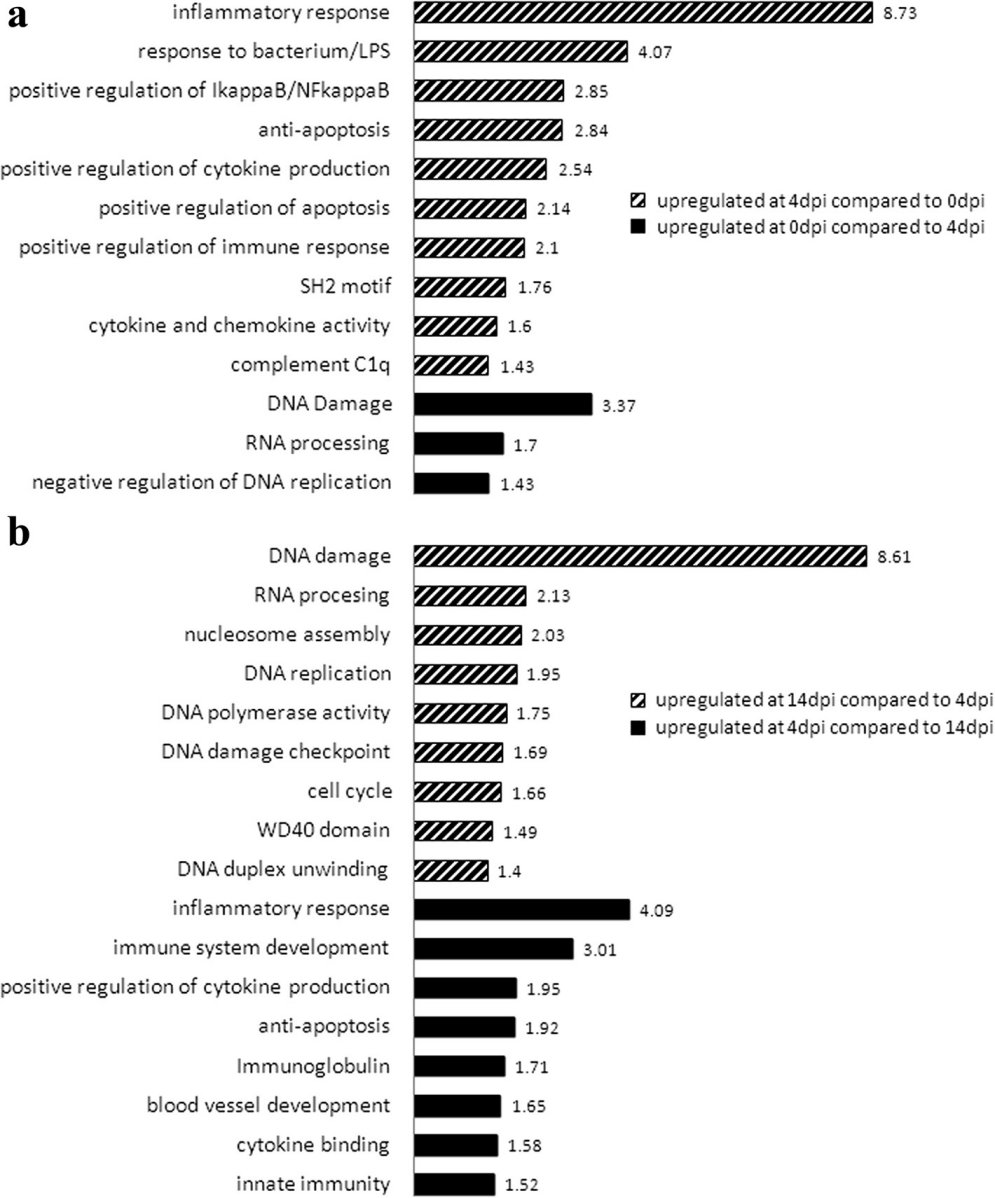
Relevant GO enrichment terms for dpi comparisons of blood RNA DE genes. Significant GO enrichment clusters are described by an explanatory name, with the enrichment score shown at the end of each bar. **a** GO enrichment between 4 dpi and 0 dpi. The striped bars are up-regulated at 4 dpi compared to 0 dpi and point to immune-related GO terms, and the black bars are down-regulated at 4 dpi compared to 0dpi and show RNA processing and DNA damage GO terms **b** GO enrichment between 14 dpi and 4 dpi. The striped bars are up-regulated at 14 dpi compared to 4 dpi and point to cell cycle and DNA damage GO terms, and the black bars are down-regulated at 14 dpi compared to 4 dpi and show immune-related GO terms

RNA of whole blood from eight littermate pairs of AA and AB weaner pigs was collected at 5 time points (0, 4, 7, 10 and 14 dpi) during a PRRSV infection. Weight gain over 42dpi, as well as viral load over 21dpi, measured as area under the log curve of viremia levels from 0 to 21 dpi, did not significantly differ between these two groups of animals (*p* = 0.54 and *p* = 0.55, respectively). Prior to RNA-seq analysis, most alpha and beta hemoglobin mRNAs were removed to increase the detection of low abundance mRNAs [14]. Paired-end RNA-seq reads were mapped to the *Sus scrofa* genome to assign reads to annotated gene coordinates and mapped reads were normalized as described [13]. For simplicity, we will refer hereafter to transcripts as the entity whose expression is being estimated by the RNA-seq data. A repeated measures linear model was used to estimate the effects of WUR genotype, day, and genotype-by-day interactions. To identify DE transcripts across time, a false discovery rate (FDR) of 5 % was used for all possible pairwise time point comparisons. A total of 3,511 transcripts were found to be DE among all time points. The number of DE transcripts ranged from 117 in the 14/10 dpi comparison to 1,991 in the 14/4 dpi comparison (Table 1). Gene ontology (GO) term enrichment analysis was performed on all 10 comparisons (Additional file 1: Table S1). Since the DAVID and Gorilla GO annotation tool gave similar results, only DAVID annotations and Enrichment scores are shown. Two informative comparisons were the list of transcripts that were DE between 4 dpi and 0 dpi and between 14 dpi and 4 dpi. GO enrichment clearly demonstrates that early after infection inflammatory response functions were induced, followed by a switch to DNA damage response, cell cycle checkpoints and DNA replication (Fig. 1).

**Table 1:** Number of transcripts compared at each day post PRRSV infection, regardless of WUR genotype

# of DE transcripts based on day effect (FDR < 0.05)			
4/0 dpi	7/0 dpi	10/0 dpi	14/0 dpi
1519	356	321	481
	7/4 dpi	10/4 dpi	14/4 dpi
	422	1025	1991
		10/7 dpi	14/7 dpi
		127	1026
			14/10 dpi
			117

**Fig. 2:**
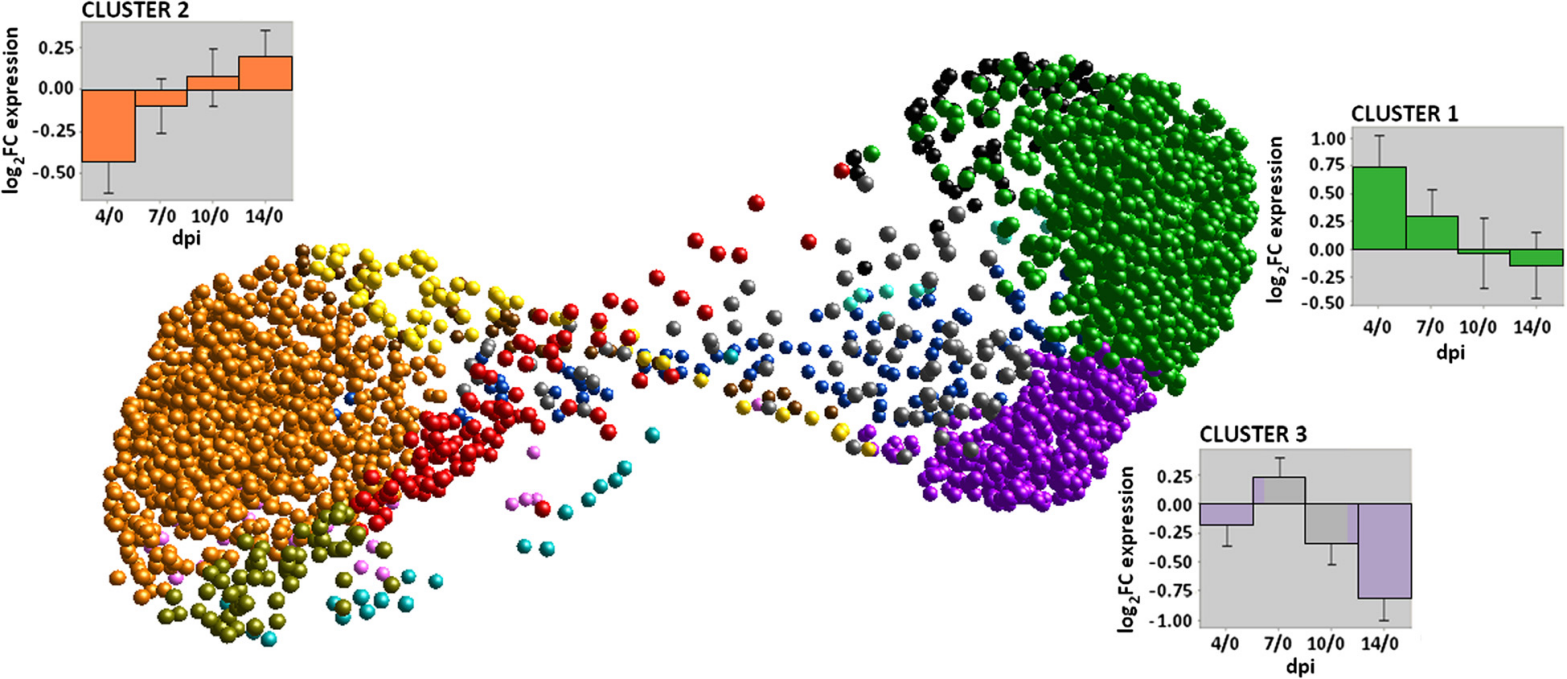
BioLayout Express^3D^ (BE3D) clustering of 3,511 transcripts that were DE over time post PRRSV infection. These transcripts were DE at an FDR < 0.05 in at least one of the 10 pairwise time point comparisons. A total of 13 clusters were formed. The histograms represent the expression patterns over time of the three largest clusters (green, orange and purple) that had enough nodes to perform a GO term enrichment analysis

To explore these results in more detail, BioLayout Express^3D^ (BE3D) was used to visualize and cluster all 3,511 transcripts that were DE across dpi (Fig. 2). The histograms shown represent the expression patterns over time of the three largest clusters. The numbers on the Y-axis correspond to the log_2_ fold change (FC) of 4, 7, 10 and 14 dpi compared to 0 dpi. Cluster 1 contained 1,206 nodes and showed greater expression at 4 dpi compared to 0 dpi, which then decreased at later dpi. Annotation of this cluster indicated enrichment of transcripts involved in inflammatory response, immune system development, anti-apoptosis, and cytokine and chemokine activity.

The second largest cluster, cluster 2, contained 1,181 nodes and was down-regulated at 4 dpi, but increased in expression over time and showed an up-regulated expression at 14 dpi. GO terms such as ‘DNA repair’, ‘DNA damage checkpoint’, ‘nucleotide excision repair’, ‘DNA packaging’, ‘mitosis’, and ‘ncRNA and rRNA processing’ were prominent in enrichment analysis of this cluster. Thus, this clustering method verified and further specified the expression pattern of transcripts responsible for the inflammatory immune response that was highly expressed early after infection (cluster 1), as well as the set of transcripts representing DNA repair functions (cluster 2) identified in the full DE dataset GO annotation shown in Additional file 1: Table S1 and Fig. 1.

A third cluster, with 516 nodes, was up-regulated at 7 dpi compared to 0 dpi but down-regulated at all other dpi compared to 0 dpi and showed an enrichment of terms such as ‘G-protein coupled receptor’, ‘synaptic transmission’, ‘ion homeostasis’ and ‘ion channel complex’ (cluster 3). Next, these clusters were further analyzed by contrasting their expression between genotypes.

### Identification and annotation of DE transcripts between WUR genotypes

An analysis of differences in transcript expression between animals with AA versus AB genotype at the WUR SNP could help unravel this large genotype effect on piglet growth and viral load post-infection. Thus, transcript expression differences between AA and AB animals were compared within each day or averaged over all days. Even at an FDR of 10 %, relatively few DE transcripts were identified, ranging from only 2 DE transcripts between AA and AB animals at 4 dpi, to 88 transcripts at 10 dpi (Table 2) and no enriched GO terms were found using DAVID analysis (data not shown). Examining all 1,370 genes with a genotype effect or a genotype x day interaction effect revealed GO enrichment of “cytoplasmic vesicle” (*p* < 0.004), “regulation of mitogen-activated protein kinase (MAPK) activity” (*p* < 0.02) and apoptosis (*p* < 0.04) were revealed (data not shown).

**Table 2:** Number of transcripts compared between WUR genotypes on a given day or averaged over all days

# of DE transcripts based on genotype effect (FDR < 0.10)					
AA vs AB, 0 dpi	AA vs AB, 4 dpi	AA vs AB, 7 dpi	AA vs AB, 10 dpi	AA vs AB, 14 dpi	AA vs AB, avg
20	2	55	88	58	33

**Fig. 3:**
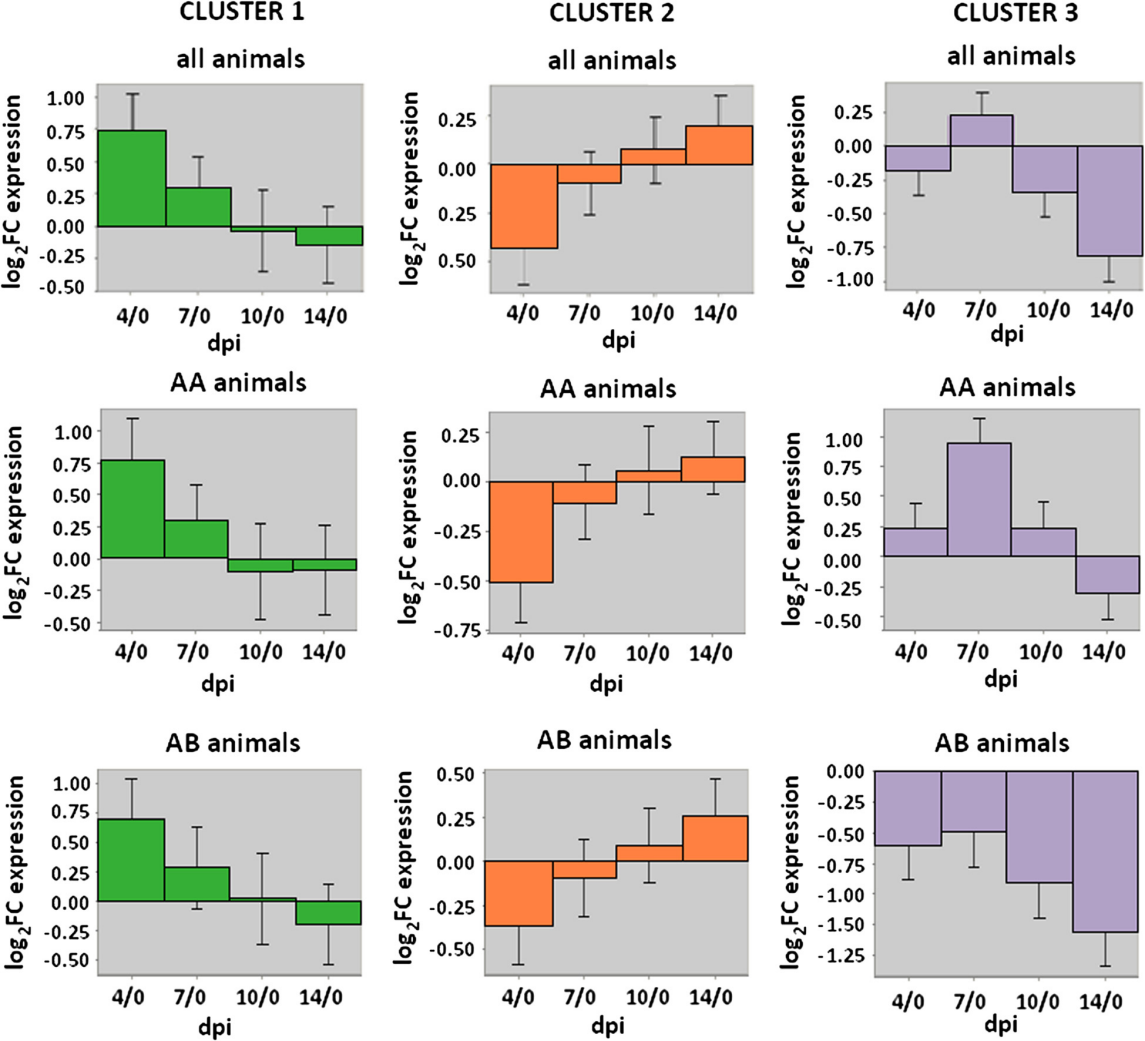
Expression over time of blood RNA transcripts in the largest BE3D clusters. Clusters shown are based on all animals or by WUR genotype. Histograms for combined genotypes are repeated from Fig. 3 for ease of comparison

**Fig. 4:**
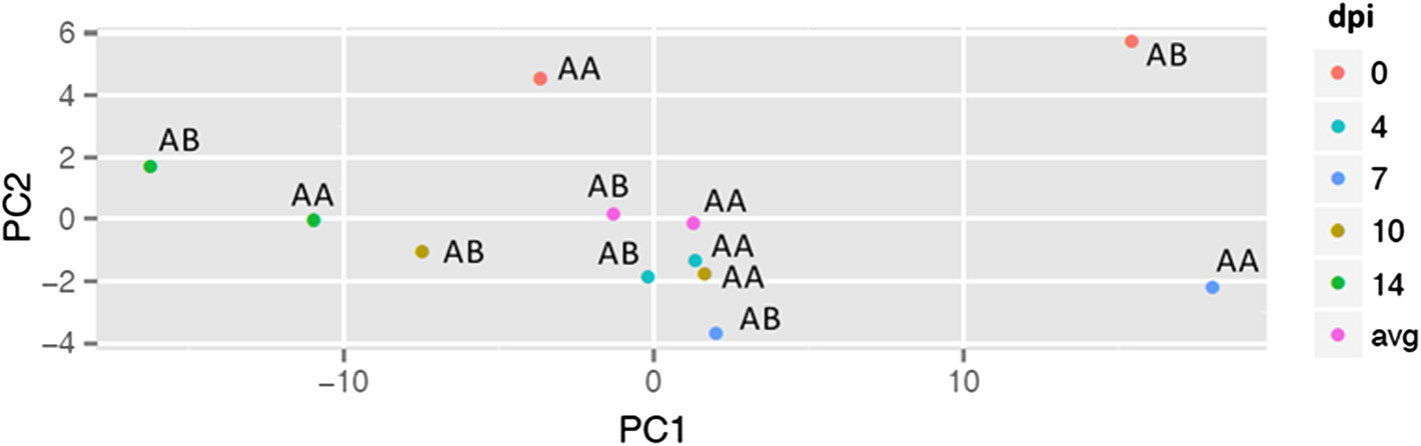
PCA plot for 516 transcripts of the BE3D cluster 3. The PCA plot is created using the model means for AA and AB on each dpi or averaged across days. The first principal component (PC1) explained 75.2 % of the variance, the second principal component (PC2) explained 6.2 % of the variance. The largest difference between AA and AB animals was at 0 dpi and 7 dpi

Expression pattern differences between pigs with alternate WUR genotypes could also be found for the three BE3D clusters described earlier (Fig. 3). AA and AB animals showed no significant difference in average expression for cluster 1 transcripts (*p* = 0.64); AB animals showed an overall higher average expression for the cluster 2 transcripts, however these were not significantly different either (*p* = 0.11). Remarkably, however, the average expression of cluster 3 transcripts was significantly different between AA and AB animals (*p* = 8e-4). The AB animals down-regulated the cluster 3 transcripts immediately after infection, while AA animals showed first an elevation up till 7 dpi, after which a decrease in expression was noticed. When examining differences between genotypes for cluster 3 transcripts on separate days rather than log_2_FC comparisons with 0 dpi, it became clear that differences between genotypes were greatest at 0 dpi (higher in AB animals; *p*-value = 0.07) and 7 dpi (higher in AA animals; *p*-value = 0.07), but were not significant at 4 dpi (*p*-value = 0.88), 10 dpi (*p*-value = 0.52) and 14 dpi (*p*-value = 0.30). Principal Component Analysis (PCA) of data from individual dpi revealed that differential expression between AA and AB for these transcripts was already present at 0 dpi (Fig. 4). Further, the relative positions of AA and AB were reversed for all other dpi, indicating that genotype-specific expression patterns captured by principal component 1 (PC1), which explained over 75 % of the transcripts expression variance among samples, changed dramatically after day 0.

### Validation of BE3D cluster 3 with independent RNA-seq data

The log_2_FC of the 516 transcripts of cluster 3 at 4, 7, 10 and 14 dpi compared to 0 dpi of PHGC3 were plotted against the log_2_FC values for those transcripts for the same dpi contrasts in similar blood samples collected from 16 AA and 5 AB animals in the PHGC5 RNA-seq study (Additional file 1: Table S2, Additional file 2: Figure S1). Correlation coefficients were 0.39 (*p*-value = 4.2e-20), 0.31 (*p*-value = 4.1e-13), 0.23 (*p*-value = 1.3e-07) and 0.17 (*p*-value = 1.1e-4) for 4/0, 7/0, 10-11/0 and 14/0 dpi respectively.

### Verification of BE3D transcript clusters using Weighted Gene Co-expression Network Analysis (WGCNA)

We used another clustering approach to verify that these clusters were robust and to further explore their biological meaning. Using WGCNA on datasets comparing 4 dpi, 7 dpi, 10 dpi and 14 dpi with 0 dpi and on datasets examining individual days (0 dpi, 4 dpi, 7 dpi, 10 dpi and 14 dpi), we found several modules whose eigengene was associated with WUR genotype. The eigengene of a module is defined as the eigenvector associated with the first principal component of the expression matrix and is used as a linear combination of expression from all genes in the module [15]. Significant modules showing interesting GO enrichment are listed in Table 3 (datasets 4/0 dpi, 7/0 dpi, 10/0 dpi and 14/0 dpi) and Table 4 (datasets 0 dpi, 4 dpi, 7 dpi, 10 dpi and 14 dpi), together with the number of transcripts in that module, their association with WUR genotype, quantified by the correlation coefficient with WUR code (0/1), and the nominal p-value for this association. When comparing GO term enrichment, the results of these analyses were consistent with the BE3D clustering results shown in Fig. 3. In Table 3, ‘SH2 domain, B, T, NK cell signaling pathways’ is a GO term enriched in the fifth significant module in the 4/0 dpi dataset, with greater expression in the AA animals, while in Fig. 3, expression of cluster 1, annotated with immune response GO terms, is also slightly greater in the AA animals compared to the AB animals at day 4 compared to 0. Similarly, the first and third significant modules in the 4/0 dpi contrast, illustrating GO terms such as ‘mitosis’, ‘DNA damage checkpoint’ and ‘DNA repair’, were elevated in the AB animals (Table 3); in Fig. 3, at 4/0 dpi, the AB animals have greater expression of cluster 2, which was annotated for DNA repair. GO terms such as ‘GPCR signaling pathway’, ‘ion transport’ and ‘ion homeostasis’ were enriched in WGCNA modules in the 4/0 dpi, 7/0 dpi and 14/0 dpi dataset with greater expression in the AA animals (Table 3), and the expression of cluster 3, annotated for these GO terms, was significantly higher in the AA animals at all time points after infection.

**Table 3:**
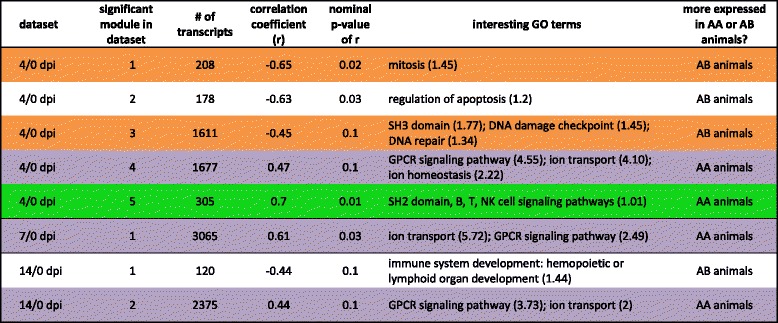
Overview of modules of DE genes whose eigengene is significantly correlated with WUR genotype

The table here presented shows immune-related GO enrichment across time-points. For 4/0 dpi, 7/0 dpi, 10/0 dpi and 14/0 dpi comparisons the significant modules (p ≤ 0.10) are listed together with the number of transcripts, the correlation coefficient with WUR genotype, the nominal p-value for that correlation and the interesting Gene Ontology (GO) terms, with enrichment scores in brackets. Positive correlation coefficients point to modules that contain transcripts that are more highly expressed in AA than in AB animals. Negative correlation coefficients refer to modules with transcripts that are more highly expressed in AB animals. Rows are color-coded according to their agreement with GO terms in clusters formed by BioLayout Express^3D^ (BE3D): green for cluster 1, orange for cluster 2, purple for cluster 3

**Table 4:**
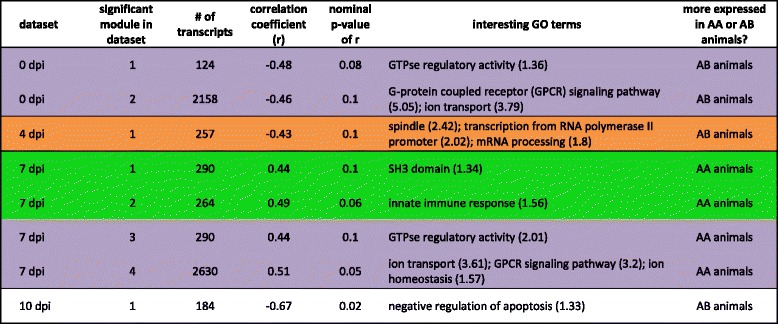
Overview of modules of DE genes whose eigengene is significantly correlated with WUR genotype

The table here presented shows immune-related GO enrichment at single time points. For 0 dpi, 4 dpi, 7 dpi, 10 dpi and 14 dpi the significant modules (p ≤ 0.10) are listed together with their number of transcripts, the correlation coefficient, the nominal p-value for that correlation and the interesting Gene Ontology (GO) terms, with enrichment scores in brackets. Positive correlation coefficients point to modules that contain transcripts that are more highly expressed in the AA animals compared to the AB animals. Negative correlation coefficients refer to modules with transcripts that are more highly expressed in the AB animals. Rows are color-coded according to their agreement with GO terms in clusters formed by BioLayout Express^3D^ (BE3D): green for cluster 1, orange for cluster 2, purple for cluster 3

Examining each dpi separately with WGCNA showed that enriched GO terms such as ‘SH3 domain’ and’innate immune response’ appeared at 7 dpi in the AA animals and GO terms such as ‘spindle’, ‘transcription from RNA polymerase II promoter’ and ‘mRNA processing’ appear in AB animals at 4 dpi (Table 4). Enriched GO terms pointing to similar functions as those enriched in cluster 3 (Fig. 3) such as ‘GTPase regulatory activity’, GPCR signaling pathway’, ‘ion transport’ and ‘ion homeostasis’ can be found at 0 dpi in AB animals and at 7 dpi in AA animals (Table 4). This indicates that AB animals, have these processes elevated before infection, relative to AA animals, after which their expression declines; whereas after infection AA animals maintain activation of such pathways. Further, compared to AB animals, this difference was most apparent at 7 dpi.

### Cell type enrichment analysis (CTEN)

**Fig. 5:**
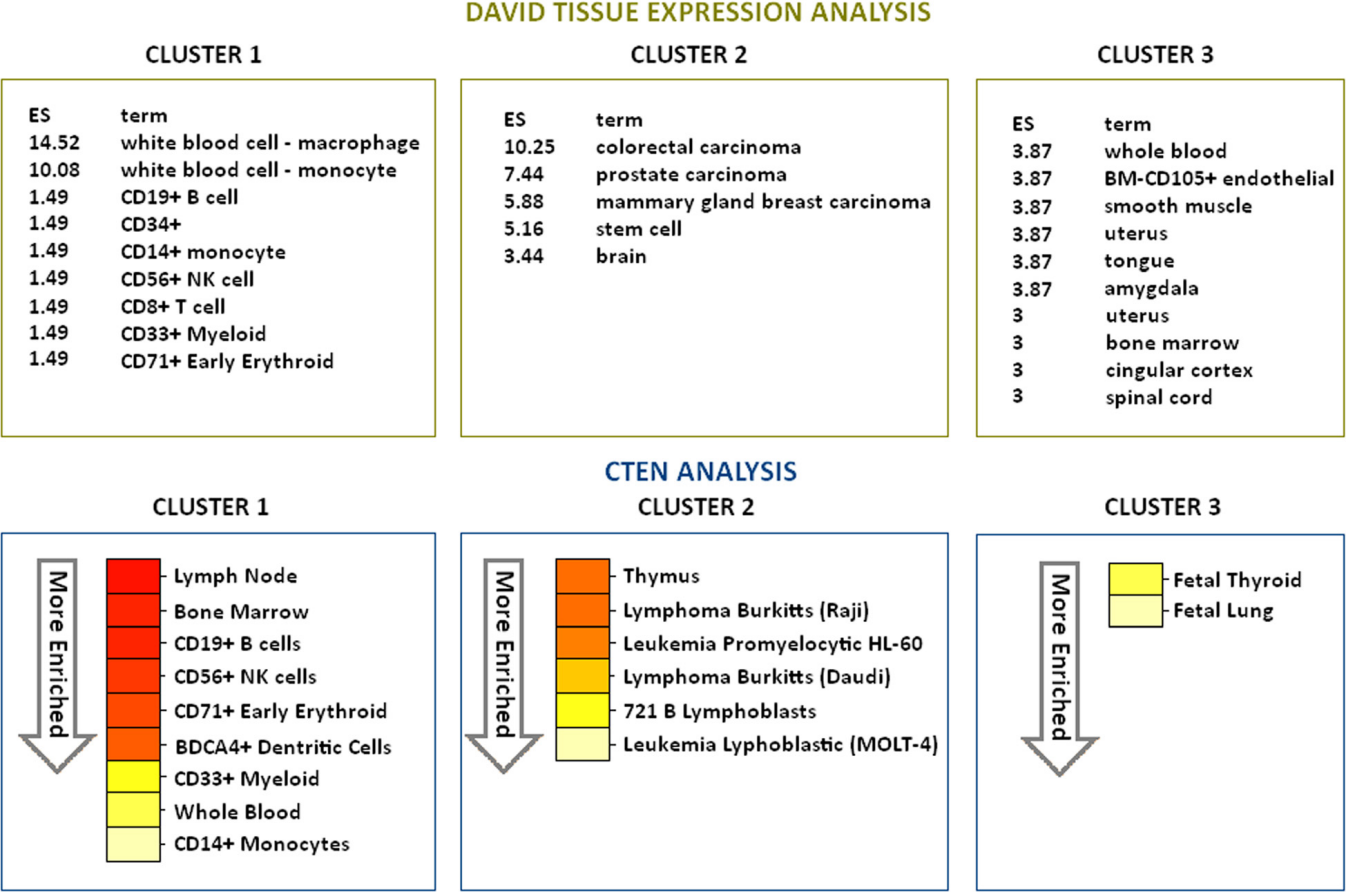
Cell type enrichment analyses of genes identified as the three largest clusters from BE3D. For DAVID, enrichment scores (ES) are shown in front of specific cell types. For CTEN, from red to light yellow are all significant enriched cell-types within each cluster transcript list (enrichment score ≥ 2.0), going from enriched to most enriched

After clustering transcripts in each dataset based on their expression patterns, both DAVID and CTEN software were used to evaluate whether these clusters represent transcripts that were usually expressed by (a) certain cell type(s). If so, a difference in expression of the cluster between AA and AB animals could indicate an engagement, or lack of, certain cell types in one of the two genotypes. The cell type enrichment analyses were applied on the three clusters formed by BE3D (Fig. 5). For both DAVID and CTEN, the most enriched cell types in cluster 1 were macrophages and monocytes, followed by myeloid cells, early erythroid cells, NK cells, B cells and T cells. This cluster represented an immune-related cluster, so it is not surprising that immune cells were enriched. Cluster 2 showed an enrichment for lymphoblasts and leukemia-lymphoma with CTEN but DAVID showed carcinoma cells and stem cell enrichment. Based on CTEN, cluster 3 was characterized by transcripts that are mainly expressed in fetal thyroid and fetal lung tissue, and the “ion transport” GO annotation of this cluster is consistent with the known importance of ion transport in these developing tissues [16–19]. DAVID analysis of cluster 3 showed very diverse cell types, illustrating that it was not easy to classify the transcripts of this cluster to a specific cell type.

### Regulatory Impact Factor (RIF) and Phenotypic Impact Factor (PIF) analyses

We were also interested in determining whether specific transcripts could be identified as important players, or “hubs”, for the differences in expression between genotypes. We analyzed the full RNA-seq dataset using RIF and PIF analyses [20]. Whereas RIF1 focuses on regulators that are differentially wired with highly abundant DE transcripts between two groups of animals (e.g., AA and AB animals), RIF2 ranks regulators that are predictors of the change in abundance of DE transcripts [20]. The PIF analysis scores transcripts high that are DE and at the same time highly abundant, since a small difference in those highly abundant transcripts could impact networks greatly [20]. A DE transcript that is not highly abundant will only have a high PIF score when it is highly DE. After imposing a |z-score| threshold of 2 to identify hubs with largest differences between genotypes in neighborhood connections, a total of 429 and 464 transcripts passed that threshold for RIF1 and RIF2 respectively, meaning that a relative large number of transcripts acted in a different way as regulator of other transcripts in their network between WUR genotypes. Table 5 and Table 6 list the RIF1 and RIF2 |z-scores| for the 10 most extreme regulators. For the PIF analysis, almost all transcripts (*n* = 8,585) passed the threshold of significance (adj. *p*-value < 0.01). The top 10 transcripts are shown in Table 7.

**Table 5:** Top 10 transcripts in both directions for the RIF1 analysis. The RIF1 analysis was performed on blood RNA DE genes contrasting AB minus AA animals.. The transcript ID, gene symbol and gene name is shown, together with the RIF1 calculated z-score. The positive RIF1 scores are > 2, which indicates a large difference between WUR genotypes in linkages to these hub genes

Transcript_id	Gene symbol	Gene	RIF1 z-score
ENSSSCT00000001129	TFAP2A	transcription factor AP-2 alpha	-0.863
ENSSSCT00000000409	MYL6	myosin, light chain 6	-0.862
ENSSSCT00000000824	LRTM2	leucine-rich repeats and transmembrane domains 2	-0.862
ENSSSCT00000002712	CATSPERB	catsper channel auxiliary subunit beta	-0.861
ENSSSCT00000000267	KRT5	keratin 5	-0.861
ENSSSCT00000000210	C1QL4	complement component 1, q subcomponent-like 4	-0.861
ENSSSCT00000002491	C15ORF55	a.k.a. NUT midline carcinoma, family member 1	-0.860
ENSSSCT00000004145	MOCOS	molybdenum cofactor sulfurase	-0.860
ENSSSCT00000000450	GPR182	G protein-coupled receptor 182	-0.860
ENSSSCT00000000014	-	Structural maintenance of chromosomes protein	-0.859
ENSSSCT00000002033	IQGAP1	IQ motif containing GTPase activating protein 1	7.348
ENSSSCT00000029413	-	uncharacterized	7.389
ENSSSCT00000012040	B4GALT1	UDP-Gal:betaGlcNAc beta 1,4- galactosyltransferase, polypeptide 1	7.530
ENSSSCT00000012873	ACTL6A	actin-like 6A	7.691
ENSSSCT00000011122	TOMM20	translocase of outer mitochondrial membrane 20 homolog	8.054
ENSSSCT00000001714	PPARD	peroxisome proliferator-activated receptor delta	8.557
ENSSSCT00000006566	ATAD2	ATPase family, AAA domain containing 2	9.040
ENSSSCT00000030357	WDR5B	WD repeat domain 5B	9.160
ENSSSCT00000005554	PSMC6	proteasome 26S subunit, ATPase, 6	9.500
ENSSSCT00000019067	NPEPPS	aminopeptidase puromycin sensitive	14.674

**Table 6:** Top 10 transcripts in both directions for the RIF2 analysis. The RIF2 analysis was performed on blood RNA DE genes contrasting AB minus AA animals. The transcript ID, gene symbol and gene name is shown, together with the RIF2 calculated z-score. The positive RIF2 scores are > 2, the negative RIF2 scores are < -2, and thus all genes presented here are highly significant, which indicates a large difference between WUR genotypes in linkages to these hub genes

Transcript_id	Gene symbol	Gene	RIF2 z-score
ENSSSCT00000005054	-	uncharacterized	-12.312
ENSSSCT00000002033	IQGAP1	IQ motif containing GTPase activating protein 1	-10.874
ENSSSCT00000007586	CCBL2	cysteine conjugate-beta lyase 2	-10.365
ENSSSCT00000004284	EPS15	epidermal growth factor receptor pathway substrate 15	-8.242
ENSSSCT00000002542	FUT8	fucosyltransferase 8 (alpha (1,6) fucosyltransferase)	-7.621
ENSSSCT00000005334	UBE3A	ubiquitin protein ligase E3A	-7.528
ENSSSCT00000007421	CAPZA1	capping protein (actin filament) muscle Z-line, alpha 1	-7.337
ENSSSCT00000007964	DYNLRB1	dynein, light chain, roadblock-type 1	-7.336
ENSSSCT00000010053	RG9MTD2	tRNA methyltransferase 10 homolog A	-7.285
ENSSSCT00000001175	CDKAL1	CDK5 regulatory subunit associated protein 1-like 1	-6.995
ENSSSCT00000002288	-	40S ribosomal protein S3a	7.188
ENSSSCT00000004971	ATP5A1	ATP synthase, H+ transporting, mitochondrial F1 complex, alpha subunit 1	7.207
ENSSSCT00000009172	CCT4	chaperonin containing TCP1, subunit 4 (delta)	7.218
ENSSSCT00000006787	TRAM1	translocation associated membrane protein 1	7.360
ENSSSCT00000004039	SF3A3	splicing factor 3a, subunit 3, 60 kDa	7.966
ENSSSCT00000000103	TOMM22	translocase of outer mitochondrial membrane 20 homolog	8.628
ENSSSCT00000005108	LEO1	Paf1/RNA polymerase II complex component	8.708
ENSSSCT00000002242	PSME2	proteasome activator subunit 2 (PA28 beta)	9.040
ENSSSCT00000002808	RPS21	ribosomal protein S21	11.614
ENSSSCT00000004960	EF1ALPHA	eukaryotic translation elongation factor 1 alpha 1	11.644

**Table 7:** Top 10 transcripts for the PIF analysis based on adjusted p-value. The transcript ID, gene symbol and gene are shown, together with the adjusted p-value and the PIF score

Transcript_id	Gene symbol	Gene	adj.P.Val	PIF.value
ENSSSCT00000025260	ACTB	beta actin	5.11E-93	136.357
ENSSSCT00000031794	EEF2	eukaryotic translation elongation factor 2	1.25E-93	118.423
ENSSSCT00000006982	ARHGAP30	Rho GTPase-Activating Protein 30	9.51E-96	103.526
ENSSSCT00000031940	CALM1	calmodulin 1	3.94E-96	96.954
ENSSSCT00000013301	SH3KBP1	SH3-domain kinase binding protein 1	2.29E-94	74.865
ENSSSCT00000024405	PSMD2	proteasome 26S subunit, non-ATPase, 2	7.17E-99	73.512
ENSSSCT00000003036	AP1G1	AP-1 complex subunit gamma-1	2.20E-100	67.972
ENSSSCT00000018259	VPS41	vacuolar protein sorting 41	4.95E-96	64.869
ENSSSCT00000015192	C19ORF50	a.k.a. KxDL Motif-Containing Protein 1	2.40E-93	61.293
ENSSSCT00000019336	KIAA0100	breast Cancer Overexpressed Gene 1	2.13E-94	58.151

*IQGAP1* was found in both the top RIF1 and RIF2 lists. IQGAP1 binds calmodulin [21–23] and *CALM1*, which encodes the protein calmodulin, had one of the highest PIF scores. Other transcripts in these extreme RIF and PIF lists point to actin cytoskeleton forming processes (*ACTB* [24], *ACTL6A*, *ARHGAP30* [25], *CAPZA1* [26], *CCT4* [27], *DYNLRB1* [28]) as well as to ubiquitin/proteosomal degradation (*PSMC6*, *PSMD2*, *PSME2*, *UBE3A*), transcriptional and translational regulation (*LEO1* [29], *EF1ALPHA*, *EEF2*, *RPS21*), and endosomal trafficking (*EPS15* [30, 31], *VPS41* [32], *c19orf50* [33], *AP1G1* [34]).

## Discussion

The goal of this study was to identify key transcriptomic differences between AA and AB animals in order to understand why AB animals respond better to a PRRSV infection. Analysis using a linear model indicated only minor differences in expression patterns between these AA and AB animals over time. However, further analyses showed a slightly but not significantly higher expression of transcripts early after infection involved in general immune response in the susceptible AA animals compared to resistant AB animals, most likely evoked by monocytes, as indicated by the CTEN analysis. More substantial was the difference in expression of transcripts with DNA damage repair GO terms, with higher expression of transcripts involved in DNA repair in the AB animals, especially early after infection. Cluster 2 showed an enrichment for lymphoblasts and leukemia-lymphoma, carcinoma cells and stem cell enrichment. Gene expression studies in lymphoblastic leukemia patients report involvement of DNA repair and DNA replication genes [35, 36]. Stem cells need to differentiate, especially during an immune response, which is consistent with the GO terms found for cluster 2. However, the most significant differences in transcript expression between the two WUR genotypes were higher expression at 7 dpi in AA pigs for transcripts with GO terms for the G-protein coupled receptor (GPCR) pathway and ion transport, including for the more specific GO terms ‘calcium’ ion transport and ‘calcium’ homeostasis, and higher expression at 0 dpi for AB animals for transcripts with similar functions (Table 4). These patterns indicate greater involvement of the GPCR pathway and calcium ion transport/homeostasis in AA animals in response to a PRRSV infection, or a higher contribution of these pathways in AB animals prior to infection, i.e., at 0 dpi. In Fig. 4, the PCA results support this hypothesis, as the largest differences between genotypes were found at 0 and 7 dpi, with the polarity of the PC1 difference between genotypes reversing between 0 and 7 dpi. AA animals at 7 dpi most closely resemble the AB animals at 0 dpi, which could be interpreted as a delayed activation of the cluster 3 transcripts until 7 dpi in the AA animals in comparison to the AB animals, who already express the cluster 3 transcripts at high levels before infection. In AA animals, a down-regulation of these cluster 3 transcripts starts after 7 dpi, while in AB animals this down-regulation initiates soon after 0 dpi.

GPCRs are known to activate phosphoinositide 3 kinase (PI3K) and PI3K phosphorylates PIP_2_ to PIP_3_, which leads to activation of Akt [37]. It has been reported that the PI3K-Akt signal transduction pathway is involved in PRRSV entry [38, 39], and other viruses also make use of the PI3K pathway to enter the cells [40–42]. With PRRS, anti-apoptosis is often seen in the early stage of infection, and macrophages die by apoptosis only later [43]. It has been proposed that the PI3K-Akt pathway is used by the virus to activate Akt, which in turn phosphorylates pro-apoptotic proteins such as Bad, caspase 9 and glycogen synthase kinase 3 beta (GSK-3β). Upon phosphorylation, these pro-apoptotic proteins are inactivated and apoptosis is delayed, allowing a short-term cellular survival during the initial stage of viral infection in favor of viral replication [44]. Since this PI3K-Akt pathway appears to be activated longer after infection in AA animals compared to AB animals, the virus may have a greater opportunity to infect cells and replicate in these animals, which makes them more susceptible to PRRS.

Ma et al. [45] reported cytoskeletal reorganization by G-coupled protein receptors dependent on PI3K, a guanosine exchange factor (GEF), and Rac1. PIP_3_ is involved in actin polymerization [46] and our results support evidence of its involvement by a multitude of actin-related transcripts that are significantly differentially regulated between AA and AB, based on the RIF analyses. Insall and Weiner (2001) hypothesized that PIP_3_ stimulates actin polymerization by recruitment and activation of Rho GTPases Rac1 and Cdc42.

IQGAP is highly differentially wired, as indicated by a high RIF score when comparing AA with AB animals, meaning that it regulates a considerable amount of transcripts differently. This gene is a GTPase activating protein that binds calmodulin, a calcium binding protein that was identified as a high PIF transcript when comparing AA with AB animals [21–23], and is found to be important in cell migration through its function in actin polymerization [47]. IQGAP is a scaffolding protein for Rac1 and Cdc42 [48] and the actin binding activity of IQGAP1 is believed to be regulated by calmodulin [49]. In addition, IQGAP1 has been found to play a pivotal interactive role in several viral attack strategies [50, 51].

Additional transcripts showing differential wiring between AA and AB animals in the RIF analysis were involved in the endosomal trafficking, which is important for viral entry [30, 52], and in the ubiquitin/proteosomal degradation pathway, which is required for efficient viral replication [53, 54]. *TOMM22* had one of the highest RIF2 scores and may be important for viral survival and replication since it encodes a mitochondrial receptor for the pro-apoptotic protein BAX [55] and has been found to be a viral miRNA target through which the virus promotes cell survival [56]. All together, it seems that AA animals are more susceptible to PRRS due to increased viral entry and replication in these animals when compared to the more resistant AB animals.

Molecular details on how PRRSV and other viruses interact with the host cell indicate the importance of calcium, calmodulin, and IQGAP in a PRRSV infection. Viruses often induce host cell cycle arrest to benefit viral proliferation by making the host cell environment available for viral replication, translation and assembly [57]. PRRSV infection delays cell cycle progression at the S phase, and as a result there is an accumulation of cells at this phase [58]. Similarly, rotavirus has been shown to hijack the host cellular machinery and push cells from the G1 to the S phase [59]. Bhowmick et al. [59] noted that viral gene expression was significantly higher in experimentally induced S phase arrested cells than in G0/G1 phase arrested cells, suggesting greater rotaviral replication during the S phase. This accumulation of cells in the S phase appeared to be Ca^+2^/calmodulin pathway dependent [59]. Rotavirus infection increased intracellular Ca^+2^ concentrations [60] and the level of calmodulin was related to progression into the S phase [61]. Bhowmick et al. [59] showed that inhibition of Ca^+2^ and calmodulin inhibited this G1 to S phase transition. IQGAP1, which is regulated by calmodulin and is an important regulator of the actin cytoskeleton, accumulated in the nucleus when cells were arrested in the G1/S phase [62].

However, how AA animals rather than AB animals activate or prolong the use of the PI3K pathway and favor viral entry and replication remains a question. It has been reported that murine guanylate binding protein 2 (mGBP2), which resembles human GBP1 (hGBP1), can form a protein complex with the catalytic subunit of PI3K [63]. By binding to PI3K, mGBP2 inhibits the activation of downstream processes involving Akt and Rac1, and thus interrupts the signal transduction pathway that otherwise could have been activated by external stimuli [63]. Furthermore, the S52N mutation, a single amino acid substitution in the GTP binding domain of mGBP2, disrupts its binding with PI3K [63]. GBPs are large GTPases that coordinate the activation of G proteins by controlling the switch from an inactive GDP-bound state to an active GTP-bound state. Three GBP proteins (GBP1, GBP2 and GBP5) contain a C-terminal CAAX prenylation motif, which allows the protein to anchor to cellular membranes [64]. Mutations in the CAAX box prevent prenylation or farnesylation, which is required for the correct localization of GTPases and for the enzyme to become completely functional [65]. Itsui et al. [66] reported that the GTPase activity of human GBP1 was required for anti-viral response against the hepatitis C virus. Fellenberg et al. [67] studied an alternatively spliced *GBP5* variant in human T cell lymphomas that misses 97 amino acids at the C terminus of the protein including the CAAX box and resembles the porcine *GBP5* truncated variant of the AA genotype for the WUR SNP that was described by Koltes et al. [13]. They propose that this truncated variant plays an important role in oncogenesis, possibly due to its loss of proper GTPase activity, and therefore formation of constitutively active, and potentially oncogenic, proteins [67]. Although this has not been confirmed, we expect that the porcine GBP5 will be able to bind PI3K in a similar manner as mGBP2 or hGBP1, because of the high homology between GBP proteins. We further hypothesize that the truncated version of GBP5, as seen in the AA animals, will not be able to bind PI3K and thus not inhibit processes downstream of PI3K, thus favoring viral entry and replication. We believe this is a plausible hypothesis because our analysis of the expression data indicates extended expression of the G-protein coupled receptor (GPCR) pathway in AA animals over AB animals.

## Conclusions

The goal of this study was to elucidate transcript expression pathway differences that can explain the association between the WUR SNP and host response to PRRS. A whole blood RNA-seq experiment was performed on blood collected during PRRSV infection of carefully selected AA (unfavorable) and AB (favorable) littermates. The main difference found between WUR genotypes indicated involvement of ion transport-homeostasis and the G-coupled protein receptor necessary for the PI3K signaling pathway, which is vital for PRRSV entry. For AB animals, this pathway is already activated before infection and declines rapidly after PRRSV infection, while in AA animals, a delayed response with regard to this pathway is observed until 7 dpi, after which it turns down. The mutation in *GBP5*, which is believed to be the causal mutation for the difference in PRRS susceptibility between AA and AB animals, likely influences the activation of this PI3K signaling pathway.

## Methods

### Experimental Design

This study was conducted as part of the PHGC project and described as PHGC3 in Boddicker et al. [10]. Experimental design and details of the infection trials are described in Lunney et al. [9] and Rowland et al. [68]. Briefly, approximately 200 commercial Landrace x Large White crossbred pigs were transported at weaning age to the biosecure testing facility at Kansas State University and allocated to pens with 10 to 15 pigs per pen. All animals came from farms that were free of PRRSV, *Mycoplasma hyopneumonia*e and swine influenza virus. After a one-week acclimation, pigs were intramuscularly and intranasally infected with a known isolate of PRRSV (10^5^ TCID_50_ of NVSL 97-7985). Approximately 3 mL of whole blood samples were collected on all pigs at 0, 4, 7, 10, 14, 21, 28, 35, and 42 dpi into Tempus blood RNA tubes (Life Technologies, Carlsbad, CA, USA) and stored at -20 °C. For the RNA-seq analysis, samples up to 14dpi were analyzed for 16 selected animals, 8 pairs of littermates consisting of one pig with the AA and one pig with the AB genotype for the WUR SNP to avoid presence of hidden genetic structure not associated with the SNP.

### Ethical statement

The study was approved by the Kansas State University Institutional Animal Care and Use Committee (IACUC) under registration number 3000.

### RNA extraction and globin reduction

Total RNA was isolated using the Tempus Spin RNA Isolation Kit (Life Technologies, Carlsbad, CA, USA) according to the manufacturer’s protocol. The quantity and quality of the RNA were assessed using a ND-1000 spectrophotometer (Nano-Drop Technologies, Wilmington, DE, USA) and an Agilent 2100 Bioanalyzer (Agilent Technologies, Inc., Santa Clara, CA, USA), respectively. The globin reduction procedure was performed using RNase H with porcine specific oligonucleotides targeting hemoglobin alpha and beta mRNAs [14], and then RNA quality was determined again with the Bioanalyzer to examine the RNA integrity (RIN) change during globin reduction procedure.

### Library construction, RNA-seq and RNA-seq analysis

The RNA-seq analyses are described in more detail in Koltes et al. [13]. In short, library construction was conducted at the Iowa State University DNA facility with the TruSeq™ library kit (Illumina, Inc., San Diego, CA, USA) according to manufacturer’s protocol. Sequencing was done on an Illumina HiSeq machine using 50 cycles and the paired-end read methodology as described by the manufacturer (Illumina, Inc., San Diego, CA, USA). All samples from each pair of littermates were allocated into one lane, for a total of 8 lanes, such that litter effects were confounded with lane effects and power to detect genotype effects was maximized. Initial read processing of reads from the HiSeq machine were processed using the Illumina CASAVA (v1.8) software.

After mapping reads to the reference genome (Sscrofa 10.2) using Tophat/Bowtie2 [69, 70], a total of 70 samples were retained for DE analysis after removing samples with low RIN score, samples with no or low number of reads mapped, and samples with inconsistent sequence-based genotypes with previous SNP chip based genotypes [13]. To normalize the data, the trimmed mean of M values (TMM) normalization procedure [71] from the EdgeR package in R [72] was used to normalize transcript counts based on the full set of genome-wide counts. This procedure also adjusts for difference in library size between samples. Normalized counts were then log_2_ transformed to obtain scaled values for statistical analysis with a repeated measures linear model that included the effects of WUR genotype (2 levels, AA and AB), day (5 levels, representing 0, 4, 7, 10 and 14 dpi), and genotype-by-day interactions as class variables. Additionally and independently for each transcript, the fixed effect of litter (8 levels) and the covariates of pre- and post-globin reduction RIN and 5’-3’ transcript read skewness were considered to be included based on model selection using Aikake information criterion (AIC) comparisons to select the best model. This model selection process was repeated for 4 error model types: uncorrelated errors, AR(1) based on day, ARH(1) based on day, or an unstructured error model. The best fitting model was determined by AIC. A total of 8,997 transcripts, with a specified minimal expression level of at least 10 normalized counts across samples, were retained. Contrasts were constructed to determine expression differences between WUR genotypes within and across days. The models were developed in R using the gls function from the nlme package. Multiple testing correction was conducted using the Benjamini and Hochberg false discovery rate (FDR) [73].

### Cluster visualization using BioLayout Express^3D^ (BE3D)

BE3D was used to visualize transcript expression patterns across days of all DE transcripts [74]. A total of 3,511 transcripts were found to be DE (FDR < 0.05) in at least one of the 10 possible pairwise time point comparisons (4/0 dpi, 7/0 dpi, 10/0 dpi, 14/0 dpi, 7/4 dpi, 10/4 dpi, 14/4 dpi, 10/7 dpi, 14/7 dpi and 14/10 dpi). A Pearson correlation of 0.90 was used to create clusters with similar transcript expression patterns over time. For the clustering, the Markov Cluster Algorithm (MCL) was used [75], which resulted in 13 clusters of transcripts.

### Principal Component Analysis (PCA)

PCA was performed using the ggbiplot function from the ggbiplot package in R. The input files were the model means for AA and AB animals, at each day and averaged over all days, of the 516 transcripts of cluster 3 discovered using BE3D.

### Validation of RNA-seq results

Validation of identified DE transcripts was performed using data from an RNA-seq study conducted on 21 infected (15 AA and 6 AB animals) Duroc x Landrace/Yorkshire pigs of the 5^th^ PHGC trial. The experimental design of this trial was similar as described for PHGC3, but blood collection was performed at 0, 4, 7, 11, 14, 21, 28, 35, and 42 dpi and RNA-seq analyses were executed up to 28dpi. More details of this trial are described in Boddicker et al. [12]. To normalize the RNA-seq data, the same model selection procedure as described above for PHGC3 was used, except for not having family in the model, since piglets used for RNA-seq were not selected based on litter in this experiment. The transcripts chosen to validate were 516 transcripts of cluster 3 discovered using BE3D in the PHGC3 data. The RNA-seq PHGC5 data up to 14dpi was used to compare both datasets.

### WGCNA analysis

WGCNA is an analysis tool to cluster transcripts that have a similar expression pattern across the samples examined [76]. WGCNA was originally developed to analyze microarray data but can and is used to examine RNA-seq data as well [77, 78]. Input for the WGCNA analyses were the TMM normalized values for each transcript. First, this was done for all available samples at each individual day (0 dpi, 4 dpi, 7 dpi, 10 dpi and 14 dpi) to identify modules that had different expression between WUR genotypes. Second, analyses were performed on values created by comparing every dpi with 0 dpi (4/0 dpi, 7/0 dpi, 10/0 dpi and 14/0 dpi) on all animals that had expression data for both days. A soft threshold was chosen to create networks with a scale free topology, using the method described by Langfelder and Horvath (2008) [76]. After the networks were built, modules of transcripts with similar expression patterns are created and eigengenes for these modules are calculated. Finally, correlations between these eigengenes and the factor of interest were calculated. The factor of interest was the WUR genotype, and the genotypes were coded so that the AB animals were “0” and the AA animals were “1”. A negative (positive) correlation between module eigengene and WUR genotype therefore signifies a greater expression level of the module in the AB (AA) animals.

### GO enrichment analysis

GO terms were obtained for the DE lists between days and between genotypes on specific days, as well as for those WGCNA clusters of transcripts that were significantly different between genotypes. For this purpose, the annotation tool DAVID Bioinformatic Resources v6.7 [79] was used. In addition, lists were examined using the annotation tool Gorilla [80]. All 8,997 expressed transcripts expressed were used as a background transcript dataset for these analyses.

### Cell type enrichment analysis

Both DAVID [79] and CTEN [81] were used to investigate evidence of cell type specific enrichment, as increases or decreases of transcript expression in blood could be due to the proportion of different cell types rather than or in addition to the up- or down-regulation of transcripts expressed by one cell [81]. For this analysis, these tools consider a broad range of cell types, from tissue cells to specific immune cells in whole blood, and even cells in a particular state [81]. These analyses were performed on the clusters created by BE3D. The DAVID analysis of enrichment of specific GO terms for different cell types was performed using the tissue expression annotation libraries CGAP_SAGE_Quartile and GN_U133_Quartile. The background list used by DAVID was as described above. For CTEN, expression levels for marker genes representing specific cell types are used to estimate changes in cell numbers between dpi. The background list used by CTEN is based on the human dataset embedded in the CTEN tool. For CTEN, Benjamini-Hochberg adjusted p-values determine the significance of enriched cell types.

### RIF and PIF analyses

RIF analyses explore differential wiring of transcript networks between two groups, i.e., AA and AB animals in this study. RIF1 and RIF2 were computed as described by Hudson et al. [82]. To test all transcripts for such differential wiring between WUR genotypes, all samples from the AB group (8 animals, 5 time points) were contrasted with all samples from the AA group (8 animals, 5 time points). In order to compare RIF1 and RIF2, z-scores were calculated by subtracting the mean and dividing by the standard deviation of all RIF1 and RIF2 scores, respectively. To create RIF1, PIF scores were calculated as described by [82].

## Availability of Supporting Data

RNA-seq data was submitted to the NCBI database under the accession number PRJNA311061: http://www.ncbi.nlm.nih.gov/bioproject/PRJNA311061/.

## Additional files

Additional file 1: Table S1. GO term enrichment performed on all 10 dpi time by time comparisons. For every time by time comparison the Enrichment Score for the significant DAVID clusters as well as a description of these clusters is presented for up-regulated and down-regulated lists of transcripts with an FDR < 0.05 for the respective comparison. **Table S2.** BE3D cluster 3 transcripts log_2_FC comparisons between PHGC3 and PHGC5. PHGC3 was the current RNA-seq study and a similar study (PHGC5) was used for validation. The log_2_FC of the 516 transcripts of cluster 3 for 4, 7, 10 and 14 dpi compared to 0 dpi of PHGC3 were plotted against the log_2_FC values at 4, 7, 11 and 14 dpi compared to 0 dpi for those transcripts in the PHGC5 RNA-seq study of 15 AA and 6 AB animals. Correlation coefficients and p-values are shown at the bottom of the table. (XLSX 85 kb) Additional file 2: Figure S1. Correlation plots of BE3D cluster 3 log_2_FC values of transcripts in PHGC3 and PHGC5. PHGC3 was the current RNA-seq study and a similar study (PHGC5) was used for validation. The log_2_FC of the 516 transcripts of cluster 3 for 4, 7, 10 and 14 dpi compared to 0 dpi of PHGC3 were plotted against the log_2_FC values at 4, 7, 11 and 14 dpi compared to 0 dpi for those transcripts in the PHGC5 RNA-seq study of 15 AA and 6 AB animals. Correlation coefficients and p-values are provided. (BMP 1243 kb)

## Abbreviations

BE3D: BioLayout Express^3D^
CTEN: cell type enrichment
DAVID: database for annotation, visualization and integrated discovery
DE: differential expressed
dpi: day post infection
FC: fold change
FDR: false discovery rate
GBP5: guanylate binding protein 5
GO: Gene Ontology
GPCR: G-protein coupled receptor
IACUC: Institutional Animal Care and Use Committee
MCL: Markov Cluster Algorithm
PCA: Principal Component Analysis
PHGC: PRRS Host Genetics Consortium
PI3K: Phosphoinositide 3 kinase
PIF: Phenotypic Impact Factor
PRRSV: Porcine Reproductive and Respiratory Syndrome virus
RIF: Regulatory Impact Factor
RIN: RNA integrity number
SNP: Single Nucleotide Polymorphism
SSC4: *Sus scrofa* chromosome 4
TCID50: 50 % tissue culture infective dose
TMM: trimmed mean of M-values
WGCNA: Weighted Gene Co-expression Network Analysis
WUR: WUR10000125
