# Supplementary figures and images for: Bioinformatic analyses in early host response to Porcine Reproductive and Respiratory Syndrome virus (PRRSV) reveals pathway differences between pigs with alternate genotypes for a major host response QTL
Source: BMC Genomics. 2016 Mar 8;17:196. doi: 10.1186/s12864-016-2547-z (PMC4782518; doi:10.1186/s12864-016-2547-z)

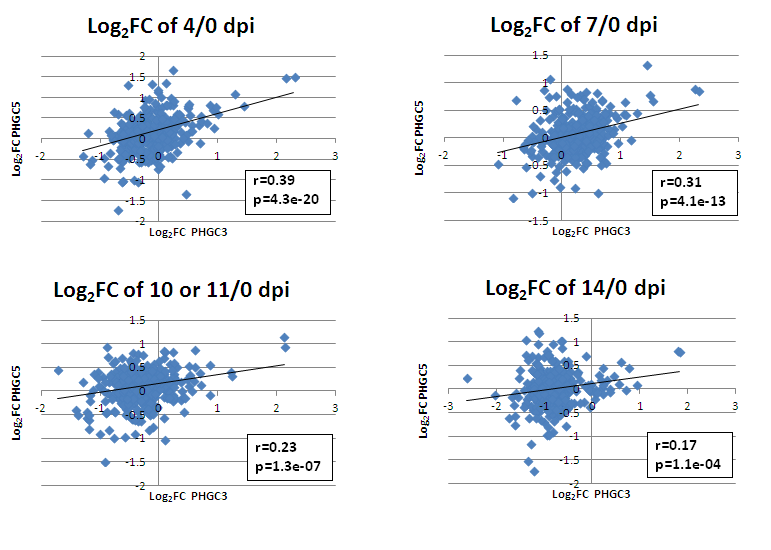

Supplement: Additional file 2: Figure S1. — Correlation plots of BE3D cluster 3 log2FC values of transcripts in PHGC3 and PHGC5. PHGC3 was the current RNA-seq study and a similar study (PHGC5) was used for validation. The log2FC of the 516 transcripts of cluster 3 for 4, 7, 10 and 14 dpi compared to 0 dpi of PHGC3 were plotted against the log2FC values at 4, 7, 11 and 14 dpi compared to 0 dpi for those transcripts in the PHGC5 RNA-seq study of 15 AA and 6 AB animals. Correlation coefficients and p-values are provided. (BMP 1243 kb) [file 12864_2016_2547_MOESM2_ESM.bmp]
